# Improving glycaemic control and life skills in adolescents with type 1 diabetes: A randomised, controlled intervention study using the Guided Self-Determination-Young method in triads of adolescents, parents and health care providers integrated into routine paediatric outpatient clinics

**DOI:** 10.1186/1471-2431-11-55

**Published:** 2011-06-14

**Authors:** Gitte R Husted, Birger Thorsteinsson, Bente Appel Esbensen, Eva Hommel, Vibeke Zoffmann

**Affiliations:** 1 The Research Department & Paediatric Ward, Hillerød Hospital, Denmark; 2 Research Unit, Department of Nursing and Health Science, Glostrup Hospital, Glostrup, Denmark; 3 Steno Diabetes Center, Gentofte, Denmark

## Abstract

**Background:**

Adolescents with type 1 diabetes face demanding challenges due to conflicting priorities between psychosocial needs and diabetes management. This conflict often results in poor glycaemic control and discord between adolescents and parents. Adolescent-parent conflicts are thus a barrier for health care providers (HCPs) to overcome in their attempts to involve both adolescents and parents in improvement of glycaemic control. Evidence-based interventions that involve all three parties (i.e., adolescents, parents and HCPs) and are integrated into routine outpatient clinic visits are lacking. The Guided Self-Determination method is proven effective in adult care and has been adapted to adolescents and parents (Guided Self-Determination-Young (GSD-Y)) for use in paediatric diabetes outpatient clinics. Our objective is to test whether GSD-Y used in routine paediatric outpatient clinic visits will reduce haemoglobin A1c (HbA1c) concentrations and improve adolescents' life skills compared with a control group.

**Methods/Design:**

Using a mixed methods design comprising a randomised controlled trial and a nested qualitative evaluation, we will recruit 68 adolescents age 13 - 18 years with type 1 diabetes (HbA1c > 8.0%) and their parents from 2 Danish hospitals and randomise into GSD-Y or control groups. During an 8-12 month period, the GSD-Y group will complete 8 outpatient GSD-Y visits, and the control group will completes an equal number of standard visits. The primary outcome is HbA1c. Secondary outcomes include the following: number of self-monitored blood glucose values and levels of autonomous motivation, involvement and autonomy support from parents, autonomy support from HCPs, perceived competence in managing diabetes, well-being, and diabetes-related problems. Primary and secondary outcomes will be evaluated within and between groups by comparing data from baseline, after completion of the visits, and again after a 6-month follow-up. To illustrate how GSD-Y influences glycaemic control and the development of life skills, 10-12 GSD-Y visits will be recorded during the intervention and analysed qualitatively together with individual interviews carried out after follow-up.

**Discussion:**

This study will provide evidence of the effectiveness of using a GSD-Y intervention with three parties on HbA1c and life skills and the feasibility of integrating the intervention into routine outpatient clinic visits.

Danish Data Association ref nr. 2008-41-2322

**Trial registration:**

ISRCTN54243636

## Background

Type 1 diabetes in adolescents is a challenge for the teenagers, their parents and the diabetes health care providers (HCPs) [1]. Despite new medical treatment modalities, the prognosis for childhood-onset type 1 diabetes remains poor [2,3]. The number of life years lost remains unchanged over the last four decades at approximately 17 years for a child diagnosed with type 1 diabetes at the age of 10 years [4]. Keeping blood glucose levels as close to normal as possible from as early in the disease as possible is known to prevent or postpone late diabetic complications [5-8]. The recommended target for haemoglobin A1c (HbA1c) in adolescents with type 1 diabetes is less than 7.5% without increasing the occurrence of hypoglycaemia [9]. However, adolescents typically do not maintain the necessary degree of diabetes self-management or the recommended HbA1c levels [10,11]. In Denmark, 31% of affected adolescents meet the recommended HbA1c threshold [12]. Although late diabetic complications are rarely seen during adolescence, there is evidence that their pathogenesis begins soon after diagnosis and accelerates during puberty [13,14].

### Challenges faced by adolescents trying to integrate diabetes into their lives

Most adolescents experience difficulties integrating the diabetes regimen into their lives; they confront significant conflicts between the need for diabetes management and psychosocial developmental needs and challenges [1,15]. Belonging to a peer group and fitting into the group's social norms and behaviours may be perceived as more important to the quality of a teenager's life than diabetes treatment [16]. Avoiding taking care of the disease as advised by HCPs and parents often leaves the adolescents with feelings of guilt, a conflicted conscience and frustration [17]. At the same time, they have conflicting experiences of being watched over, blamed and controlled by their parents [18], while also being vulnerable to the disease [19] and still needing guidance from their parents to manage the daily treatment [20-22]. This increases conflicts and deteriorates adolescent-parent collaboration and adolescent self-management [23,24]. From the adolescent's point of view, striving for independence and self-management of the disease is known to present a considerable stress [25,26].

### Challenges faced by parents in transferring responsibility

During adolescence, the responsibility for the management of diabetes should gradually be transferred from parents to adolescents [1,27]. Some parents are, however, reluctant to transfer responsibility for diabetes management, as they doubt the adolescents' abilities to self-manage their diabetes [28,29]. Other parents leave all responsibility for managing the disease to their adolescents, trying to avoid conflicts or expecting them to be competent because of their age and the amount of time since diagnosis [30]. Both approaches may lead to poor glycaemic control [31,32]. A constructive form of parental involvement comprising guidance and supervision, shared knowledge and shared responsibility yields better glycaemic control [33]. However, systematic education and guidance on how to be a constructive and supportive parent is not currently offered as part of routine care [34,35].

### Challenges faced by health care providers in their interactions with adolescents and parents

HCPs view adolescence as a difficult time in which the processes of managing diabetes, providing guidance and eliciting cooperation from adolescents and their parents are complex [36-38]. Apart from optimising medical treatment for diabetes, HCPs should aim to effectively navigate the interaction between adolescents struggling to find their identity separate from their parents and parents concerned about their child's difficulties combining teenage life with diabetes self-management [39]. HCPs should encourage parental involvement that facilitates adolescents' independent decision-making through a gradual transfer of responsibility and management of the disease [40-42]. However, current diabetes education and routine outpatient clinic visits seem to have little effect on conflict resolution, transfer of responsibility, self-management skills, and better glycaemic control [43].

### Interventions

According to Anderson [24] and Delamater [44], psychosocial and behavioural family-based controlled interventions improve self-management, glycaemic control and family relationships. However, these interventions were carried out separate from routine paediatric outpatient clinic visits. Three randomised controlled studies have partly been integrated into routine paediatric outpatient clinics [43,45,46], and two of these studies included parents (Laffel [46] and Murphy [43]). Grey and colleagues have shown that coping skills training delivered to small groups of adolescents combined with intensive diabetes management improved quality of life and glycaemic control [45]. Laffel and colleagues have shown that a family-focused teamwork intervention run by a trained research assistant increased family involvement and prevented worsening of glycaemic control [46]. Murphy and colleagues have shown potential benefits on parental involvement and glycaemic control in a structured education programme for adolescents and parents in small groups, but further studies are in progress to confirm these findings [43].

In searching for a method that could be applied by HCPs and adapted to adolescents and their parents, we chose Guided Self-Determination (GSD), which has reduced HbA1c (by 0.4%) and improved life skills in adults with persistently poor glycaemic control of type 1 diabetes [47]. We adjusted GSD to adolescents and their parents (GSD-Young, named GSD-Y hereafter) for use in paediatric diabetes outpatient clinics by the adolescents' usual HCPs. The current trial of GSD-Y is the first to evaluate the effect of an intervention involving both adolescents and parents that is carried out in routine outpatient clinics with HCPs from the adolescents' usual interdisciplinary diabetes team.

We hypothesize that using GSD-Y in routine paediatric outpatient diabetes clinics will reduce HbA1c concentrations and improve adolescents' life skills compared with those in a control group.

### Objectives

1) To test whether GSD-Y can be integrated into routine paediatric outpatient diabetes clinics in a collaboration between adolescents, their parents and the interdisciplinary diabetes HCPs.

2) To test whether GSD-Y reduces HbA1c and improves life skills in adolescents with type 1 diabetes.

3) To illustrate how GSD-Y influences developing life skills in adolescents supported by their parents and their HCPs.

## Methods/Design

### Ethical Approval

The trial will be performed in accordance with the recommendations guiding nurses in clinical research involving human participants (Helsinki Declaration). The project was reviewed by the Danish National Committee on Biomedical Research Ethics on April 17, 2009 as registry- and interview-based research (REC; reference number, 0903054 document number, 230436).

### Type of study

This study is a life-skills intervention using a mixed methods design comprised of a randomised controlled trial and a nested qualitative evaluation [48]. Objective 2 will be met through the quantitative component, whereas Objectives 1 and 3 will be met through the qualitative component. The use of a quantitative and a qualitative approach in combination increases the opportunity for a complementary evaluation, which provides a better understanding of GSD-Y's potential to influence the process of improving glycaemic control and life skills than using either approach alone. The quantitative component evaluates the effect of GSD-Y, whereas the qualitative component has two purposes: a) to elucidate the factors that affect how well GSD-Y is implemented in routine clinics and perhaps affects the outcomes, and b) to provide a detailed understanding of how GSD-Y works in triads carried out as part of routine care delivered by the adolescents' usual HCPs.

**Figure 1:**
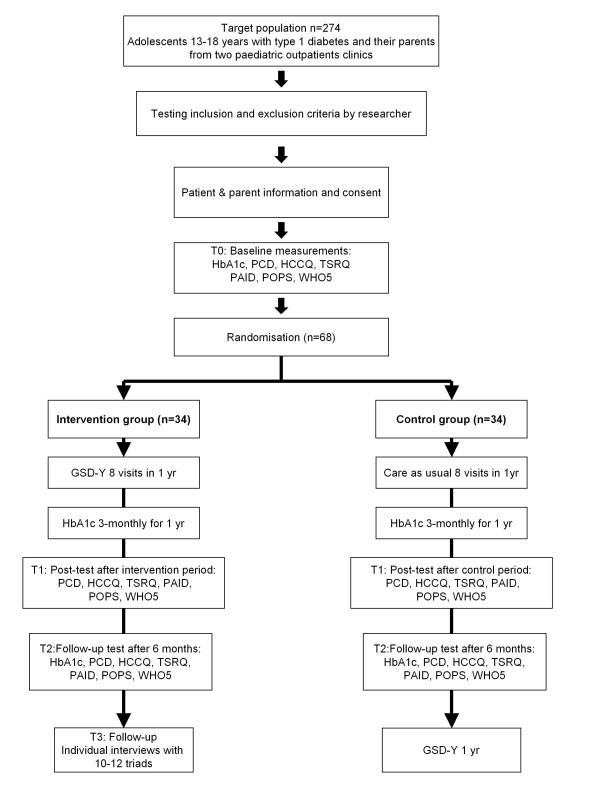
Flowchart of the study

The protocol is summarised in a flowchart (Figure 1).

### Setting

The study takes place at 2 paediatric outpatient clinics at 2 hospitals in the capital region of Denmark: Glostrup, with the largest diabetes outpatient clinic in Denmark (480 patients) and Hillerød, with the third largest diabetes outpatient clinic in Denmark (171 patients).

Two paediatric physicians, 5 paediatric diabetes nurses and 2 dieticians (HCP hereafter), trained and tested in using GSD-Y, will recruit adolescents with type 1 diabetes and conduct the GSD-Y intervention as part of their routine outpatient clinic visits.

### Guided Self-Determination-Young: theoretical and conceptual frameworks

GSD is a problem-solving and decision-making method designed to overcome barriers to empowerment in adult patient-provider interactions, and these barriers are explained by three grounded theories [49-51]. GSD has a formal theoretical foundation in life-skills theory [52,53], empowerment [54] and motivational theory of self-determination [55,56].

GSD-Y is aimed at improving glycaemic control and increasing adolescents' life skills. Life skills is defined as "those personal, social, cognitive and physical skills that enable people to control and direct their lives and develop the capacity to live with and produce change in their environment" [47,52,57]. In GSD-Y, the acquisition of life skills is considered to be a developmental process, where the adolescents are intended to start to accept and integrate diabetes into their lives and to become autonomously motivated to handle the challenges the life of a teenager with type 1 diabetes demands. To be autonomously motivated means, for example, that adolescents check their blood sugar because they find it important personally, rather than doing it on the initiative of parents or HCPs [56].

Because part of developing life skills is making self-determined decisions [52], Self-Determination Theory (SDT) has a central role in GSD-Y. According to SDT, self-determined behaviour requires the fulfilment of three needs: competence, autonomy and relatedness. An environment that is autonomy-supportive is necessary to foster the fulfilment of these needs [56]. A feeling of competence occurs when a person perceives that he or she meets optimal challenges and is able to master them effectively. Autonomy is perceived when people experience a sense of choice, endorsement and volition to act in accordance with their interests and values. The need for relatedness refers to the warmth and caring received through interactions with others, resulting in a general sense of belonging [56]. However, by applying pressure in their striving for good glycaemic control, parents and HCPs may unwillingly obstruct the adolescents' development of competence, autonomy and relatedness, potentially fostering passivity, ill-being and amotivation [58].

In our study, the adolescents' need for relatedness is satisfied when they feel a sense of belonging with parents and HCPs because there is an atmosphere where talking openly and honestly about their difficulties living with diabetes is legitimised, and adolescents still feel that they are cared for unconditionally. Satisfaction of the need for autonomy occurs when the adolescents perceive that parents and HCPs try to understand their perspective, acknowledge their feelings, and act in a non-judgmental way by minimizing the use of controlling language and behaviour. This creates an autonomy-supportive environment for decision-making, which helps adolescents act in congruence with their values and interests. Satisfying the need for relatedness and autonomy paves the way for fulfilling the need for competence in diabetes management. Adolescents will believe that they succeed in managing diabetes because parents and HCPs are aware of their current competence and accept their shifting readiness to take responsibility for their diabetes.

### Guided Self-Determination-Young: essentials in GSD-Y

GSD-Y consists of 18 semi-structured reflection sheets for adolescents (Table 1) and 5 reflection sheets for parents (Table 2). In addition, 4 new semi-structured reflection sheets have been developed for visits with dieticians (Table 3). The semi-structured reflection sheets are based on theories such as dynamic judgement building [59], values clarification [60] and the trans-theoretical stages of change theory [61], all of which enhance the development of life skills as described above.

**Table 1:** Reflection sheets for adolescents

1. visit	Your life with diabetes from beginning to now
**Reflection sheets**	Written invitation to work together in a new way
	Two ways to look at HbA1c Important events and periods in your life What do you find difficult at present living with your diabetes? Your plans for changing your way of life Agreement on things to work with till next visit

**2. visit**	**Values and opportunities**

**Reflection sheets**	Unfinished sentences: needs, values, experiences and opportunities?
	Agreement on things to work with till next visit

**3. visit**	**Diabetes in your life - now and in the future**

**Reflection sheets**	Blood sugar checks and your reasons for checking
	A picture or a metaphor, or expression describing your life with diabetes Room for your diabetes in your life Shared responsibility for your diabetes in daily life between you and your parents Agreement on things to work with till next visit

**4. visit**	**Different ways to look upon numbers**

**Reflection sheets**	Your blood-sugar numbers as you would wish them to be and as you know them from experience
	Evidence for advantages and disadvantages of high and low blood sugar Your plan for blood sugar regulation in the short and long run Situations where you want to avoid low blood sugar Agreement on things to work with till next visit

**5. visit**	**Problem-identification**

**Reflection sheets**	Current problem-solving
	Agreement on things to work with till next visit

**6. visit**	**Problem solving and options of new ways to self-management**

**Reflection sheets**	Dynamic problem-solving
	Pros and cons Agreement on things to work with till next visit

**7. visit**	**Problem-identification**

**Reflection sheets**	Current problem-solving
	Agreement on things to work with till next visit

**8. visit**	**Problem solving and options of new ways to self-management**

**Reflection sheets**	Dynamic problem-solving
	Pros and cons Solved problems and subjects to continue to work with in future outpatients appointments

**Table 2:** Reflection sheets for parents

1. visit	Your life as a parent to an adolescent with type 1 diabetes
**Reflection sheets**	Unfinished sentences: needs, values, experiences and opportunities?
	Room for your adolescents' diabetes in your life Current problem-solving

**2. visit**	**Problem identification and solving - options of new ways to shared decision making**

**Reflection sheets**	Dynamic problem-solving
	Pros and cons

**Table 3:** Reflection sheets for visits at the dietician

1. visit	Present challenges regarding food, snacks and insulin
**Reflection sheets**	What do you find demanding or difficult at present regarding your food living with your diabetes?
	Experiments: An easy situation and a difficult situation as you experience it where you try to get food/snacks and insulin to fit together

**2. visit**	**Evaluation of experiments**

**Reflection sheets**	Did it work? Why if and why if not?
	New experiments to work with till next visit or ending

Before each appointment, adolescents and parents complete the reflection sheets (Table 1, 2, 3). The first reflection sheet includes a written invitation for adolescents and their parents to take part in mutual problem solving (Table 1) [50]. While this component of the sheet clarifies that the knowledge of all three parties is important and legitimises different points of view, it also states that the adolescents are seen as the final problem solvers, and parents and HCPs are seen as facilitators.

By filling in reflection sheets with their own words and drawings, adolescents and their parents systematically explore and express their individual and common difficulties and experiences with diabetes in daily life. Thus prepared for appointments in the outpatient clinics, adolescents and their parents are guided by trained GSD-Y HCPs to communicate openly and reflect mutually by sharing and respecting each other's observations, thoughts and feelings as a starting point for a constructive collaboration in a caring relationship. This model adds shared insight to previous patterns of diabetes management, which yields a platform for identifying unknown resources in both adolescents and parents and discovering new strategies for problem-solving between the three parties. This paves the way for agreements and concrete arrangements about how to test new problem-solving strategies in the time between outpatient appointments. At the outpatient appointments, the triad evaluates their experiences with these strategies.

The overall aim is for adolescents and parents to identify concrete potential for change [47] and to avoid adolescents, parents or HCPs entering alliances with one another against the third party.

To use the reflection sheets with adolescents and parents, HCPs must be able to practice advanced communication skills such as mirroring [62], active listening [63,64] and values clarification [60]. Furthermore, HCPs should be able to support autonomy in their way of providing information and research-based knowledge of diabetes treatment and management (e.g., evidence on risks incurred by high and low blood sugar levels).

### GSD-Y training programme for HCP

To meet Objective 1, HCPs participated in a training programme (Additional file 1 ). The programme consisted of lessons in the formal theoretical basis of GSD, knowledge of barriers to empowerment in patient-provider relationships that GSD was designed to overcome and apparatuses in GSD-Y. Furthermore, they practiced using the semi-structured reflection sheets supported by their advanced communication skills using role-playing with simulated adolescents and parents, but also with real adolescents with type 1 diabetes and parents who agreed to participate in this training process. These adolescents and parents did not participate in the intervention trial. HCPs were taught and supervised by GRH and VZ. Finally, their formal theoretical foundation and ability to use GSD-Y were approved by GRH before the start of the trial.

### Endpoints of the study

#### Primary outcome

HbA1c.

#### Secondary outcomes

a. Development of life skills in adolescents with type 1 diabetes

(i) Experience of feeling competent in managing diabetes, (ii) experience of HCPs being autonomy-supportive versus controlling, (iii) motivation for diabetes management, (iv) ability to manage diabetes-related distress, (v) involvement and support for autonomy from parents, (vi) well-being.

b. Diabetes outcomes directly related to patient management

(i) Insulin delivery/number of injections/insulin types, (ii) number of self-monitored blood glucose values, (iii) hypoglycaemic episodes (frequency and severity), (iv) admissions to hospital and reasons for admissions (e.g. episodes of ketoacidosis, hypoglycaemia).

c. Diabetes outcomes indirectly related to patient management

(i) Attendance at intervention or control sessions, (ii) parental participation.

### Sample size calculation

The size of the study was based on the primary outcome measure HbA1c. According to a power calculation, an absolute difference of 1.0% in HbA1c between the GSD-Y group and the control group (power 0.8; two-sided level of significance 0.05) could be detected with 26 patients in each group. This calculation was based on a standard deviation of the HbA1c value of 1.3% from a study of coping skills training [45]. To compensate for an attrition rate of 25%, we aimed to recruit 68 patients.

### Enrolment

#### Inclusion criteria

All adolescents aged 13-18 years who have had type 1 diabetes for more than one year will be invited to participate together with their parents if they meet the following criteria:

▪ HbA1c ≥ 8.0% at the last visit before entry into the study and

▪ Average HbA1c > 7.5% during the last year before entry into the study.

#### Exclusion criteria

Adolescents will be excluded from participating in the study if they meet any of the following criteria:

▪ Diagnosed with a psychiatric disease

▪ Consulting a psychologist at the time of recruitment

▪ Unable to understand, talk or read Danish.

### Randomisation

Adolescents and parents willing to participate and fulfilling the inclusion criteria will be randomised to either an intervention group (n = 34) (GSD-Y) or a control group (n = 34) (standard care), using stratified randomisation by the adolescent's usual HCP. Randomisation will be performed using sealed envelopes. Neither adolescents nor the HCPs can possibly be blinded to the study. The adolescents in the control group will be offered the GSD-Y intervention after the study has concluded (14-18 month wait-list design).

### Consent

Consent to participate in the study will be obtained by the adolescents' usual HCP. After informed written consent is obtained from the adolescent and at least one parent, adolescents will be randomised into either the intervention or the control group. The adolescent or their parents remain free to withdraw at any time during the study without giving reasons and without prejudicing further treatment. If a participant withdraws consent from further study participation, their data will remain on file and will be included in the final study analysis if the consent for use of the data is not withdrawn; if consent for use of data is also withdrawn, data will be destroyed immediately.

### Intervention group

The GSD-Y intervention will be delivered by the adolescent's usual HCP in individual settings for a total of 8 visits during an 8- to 12 month period. Each of the 8 visits will last for 1 hour and will include specific reflection sheets, and each visit will cover a specific topic (Table 1). Parents will be invited to participate. However, at least one of the visits can take place without the parents if the involved parties agree. The purpose is to create a safe environment where the adolescents can talk about personal affairs that are confidential and not known by their parents, yet are pertinent to their ability to manage their diabetes (e.g. smoking, drugs, boy/girlfriend). After this type of visit, the adolescents and HCPs will agree on what should be told to the parents, who should tell, and when.

In addition to the visits together with their adolescents, parents will also be offered two visits alone with the adolescents' usual HCPs. The reason for this is to create an environment where the parents can talk about how to act in an autonomy-supportive manner and how to manage their adolescents' shifting readiness to take responsibility for the management of the disease. The first of these parent/HCP visits will be offered after 3 months, and the second will be offered after 6 months. Both of the visits will include specific reflection sheets, and both visits will cover a specific topic (Table 2). After these visits, the parents and HCPs will agree on what should be told to the adolescents, who should tell, and when.

Adolescents will be referred to the dietician if needed. The need for referral will be made by the adolescent, the parents and their HCPs based on the completed reflection sheets from visits 1 and 2. The meeting with the dietician can take place with or without the parents, as decided by the involved parties. Each referral to the dietician involves at least two visits. Each visit is supported by special reflection sheets and covers a specific topic (Table 3).

The adolescents and parents keep their original semi-structured reflection sheets and a copy is put in their file.

### Control group

The control group receives standard care including a number of outpatient visits equal to that of the intervention group: eight visits during an 8 to 12 month period, with a standard duration per visit of 30 to 45 minutes, depending on the hospital.

### Duration

The trial will last from 14 to 18 months for both groups including the 6-month follow-up measurements. The first adolescent began the study in September 2009, and the last adolescent will finish the study in April 2012.

### Data Collection, Measurements and Analysis Quantitative component

#### Primary outcome

HbA1c will be collected as a routine clinical measurement every third month, which is a standard practice. The capillary blood samples for HbA1c from both hospitals are being analysed at the same department of clinical biochemistry using Variant Analysis Mode, TOSOH Automated Glycohaemoglobin Analyzer HLC-723 G8 (normal range 4.3% - 5.8%).

#### Secondary outcome a

Danish versions of 6 scales were compiled in one questionnaire (Table 4). The questionnaires will be completed by the adolescents at the outpatient clinics and placed in a closed envelope before being returned to the personal HCPs at the following timepoints: 1) baseline before randomisation, 2) after the end of the intervention/control period (8-12 months), and 3) after a 6-month follow-up period (ranging between 14 and 18 months from the time of entry into the trial).

**Table 4:** Adolescent measures and outcome

**Scales**	**Outcome**	**Ranging**	**Examples**	**Scores**
The Perceived Competence Scale (PCD) 5-item	Experience of own competence	Ranging from 1 (strongly disagree) to 7 (strongly agree)	*"I feel confident in my ability to manage my diabetes"*	Produces a total sum score from 5- 35. A high sum score represents a high level of perceived competence

The Health-Care Climate Questionnaire (HCCQ) 5 -item	Perceptions of autonomy support from HCP	Ranging from 1 (strongly disagree) to 7 (strongly agree)	*"I feel that my HCPs have provided me choices and options about handling my diabetes"*	Produces a total sum score from 5-35. A high sum score represents a high level of perceived autonomy support

The Treatment Self-Regulation Questionnaire (TSRQ) 21-item Consists of 3 subscales	The degree in which patients' behaviour is self-determined	Ranging from 1 (strongly disagree) to 7 (strongly agree)	*(I) Autonomous; "It's exciting to try to keep my blood sugar in a healthy range"* *(II) Controlled; "I want my HCP to think I am a good patient"* (III) A-motivated; "I do not know why I do try - I will not bee successful"	Produces sum scores for each of three subscales, Autonomous from 8-56, Controlled 9-63, Amotivated 4-28. High sum scores indicate high levels of autonomy, controlled or amotivated behaviour. A Relative Autonomy Index is calculates by subtracting the controlled scores from the autonomous scores. The higher relative autonomy index the higher is motivation based on autonomy compared to control

The Problem Areas In Diabetes scale (PAID) 20-item	Perception of current emotional burden of diabetes related issues	Ranging from 0 (not a problem) to 4 (serious problem)	*"Feelings of guilt or anxiety when you get off track with your diabetes management"*	Produces a total score from 0-100 by summing up and multiplying this sum by 1.25. Higher scores indicate greater emotional distress. Cut points: ≥30 elevated distress ≥40 serious distress

The Perception of Parents Scale (POPS) 26-item Consists of 2 sub-scales, mothers & fathers	Perception of autonomy support and involvement from parents	Ranging from 1 (not at all true) to 7 (very true)	*(I) Mother/Father Autonomy Support;* *"My mother/father allows me to decide things for myself"* (II)Mother/Father Involvement; "My mother/father finds time to talk with me"	Produces a total sum score from 13-91 in each subscale. High sum scores represent a high level of mother/father autonomy support/involvement

The WHO5 Well-Being Index 5-item	Emotional Well-being	Ranging from 0 (not present) to 5 (constantly present).	*"I have felt cheerful and in* *good spirits for the last* *two weeks"*	Produces a total score from 0-100 by summing up and multiplying a sum score by 4. Higher scores indicate greater emotional distress. Cut points: < 50 poor emotional well-being ≤ 28 indicate depression

The scales included the following:

▪ Perceived competence for diabetes management (PCD), assessing patients' experiences of feeling able to manage their diabetes successfully [65]

▪ Health Care Climate Questionnaire (HCCQ) assessing the degree to which patients believed their HCPs to be autonomy-supportive versus controlling in providing general treatment [65]

▪ Treatment Self-Regulation Questionnaire (TSRQ) assessing the motivation for diabetes management and the degree to which behaviours tended to be self-determined. The TRSQ consists of three subscales; (I) Autonomous, (II) Controlled, (III) A-motivated [66]

▪ Problem Areas In Diabetes (PAID) assessing diabetes-related distress including a wide range of feelings related to living with diabetes and its treatment, including guilt, anger, depressed mood and fear [67]

▪ The Perception of Parents Scale (POPS) [68] assessing adolescents' perceptions of their parents' autonomy support and involvement

▪ WHO-5 Well-being Index capturing emotional well-being in the last two weeks (WHO-5) [69].

The scales were translated and harmonised in accordance with recommended guidelines [70]. Internal consistency was measured for all 6 scales and proved to be good. Cronbach's α ranged from 0.76-0.94 for the Danish versions of the HCCQ, PCD and TSRQ for adults, [47]; the Cronbach's α for the English version for adolescents of the WHO5 was 0.82 [69], 0.96 for the PAID [71] and 0.88 for the POPS Autonomy support from mothers and fathers [58]. Face validity of the Danish versions was tested in 8 adolescents between 13 and 18 years of age with type 1 diabetes.

#### Secondary outcomes b and c

Regarding secondary outcomes b and c, a case report form will be completed at every outpatient visit by the adolescents' HCPs. Furthermore, demographic data will be collected at baseline, after the intervention/control period and at the 6-month follow-up.

### Analysis

To meet Objective 2 and test if GSD-Y effectively reduces HbA1c and improves life skills in adolescents with type 1 diabetes, we will analyse HbA1c and quantitative data from the questionnaires using PAWS Statistics18 for Windows (SPSS Chicago, IL, USA). Statistical analyses will include frequency, mean, standard deviation and confidence intervals. Comparisons of primary and secondary outcomes for the two groups will be conducted comparing data at baseline, at the end of the study, and after a 6-month follow-up period using appropriate parametric tests for variables fulfilling the normal distribution criteria or appropriate non-parametric tests for variables not fulfilling the normal distribution criteria. A Bonferroni correction for multiple testing will be performed.

Improvement of life skills will be defined as increases in HCCQ-scores, TSRQ-scores on autonomy or in relative autonomy index (formed by subtracting TSRQ-scores on control from TSRQ-scores on autonomy), PCD, POPS, WHO-5 and frequency of SMBG per week, and decreases in TSRQ-scores on amotivation, PAID scores and HbA1c. Differences within the GSD-Y group and between the GSD-Y group and the control group will be calculated at the end of the intervention (8-12 months) and after a 6-month follow-up period.

### Qualitative component

#### Data collection

Ten to twelve adolescents from the intervention group and their parents and HCPs will be followed during the intervention period. To ensure that we follow triads who face significant challenges, we will select them on the basis of high PAID scores and low WHO-5 scores at baseline, which indicate difficulties with life skills.

Data will be collected during the intervention period by recording two or three outpatient appointments between 1) adolescent, parent and HCP, 2) adolescent and HCP, and 3) parent and HCP.

Individual interviews will be carried out and recorded with the above-mentioned triads after the intervention's endpoint measures at a 6-month follow-up visit using a semi-structured interview guide [72] prepared on the basis of both listening to the recordings from outpatient visits and the definition of life skills [57,73].

#### Parameters and analysis

To meet Objective 1, the analysis of the recorded outpatient visits and the individual interviews will explore how adolescents, parents and HCPs experience the following:

▪ the implementation of GSD-Y in routine clinics (e.g. appropriateness, feasibility, the triads' receptiveness, factors affecting implementation)

▪ usefulness of components of GSD-Y and additional support required for sustained uptake

▪ sustainability of GSD-Y and issues to consider in extending the model to adolescents in general with diabetes or other chronic disorders

To meet Objective 3, the analysis will also explore and illustrate how GSD-Y influences the process of adolescents developing life skills supported by their parents and their HCPs. Because the intervention is theory-driven [74], the analytical framework is predominantly deductive, based on theories on life skills [57,73], self-determination theory [56], empowerment [54], values clarification [60], Zoffmann's grounded theories [49-51] and the way we expect these skills to be recognized in the interactions between adolescents, parents and HCPs in the qualitative evaluation as operationally described below. However, the analysis will also be inductive in its use of the constant comparative method and theoretical sampling [75] to expand the existing GSD theory to build a cumulative body of theory because the evaluation of GSD-Y is the first to evaluate a version involving three parties.

Data from the recorded outpatient clinics and the individual interviews will be transcribed verbatim. NVivo 8 software will be used to facilitate the analysis. To maximise the validity of our findings, at least two researchers will participate in the analysis.

Improvement of life skills after participating in the intervention group will be defined if we recognize that the adolescents have met the following benchmarks:

- start to integrate the disease into their lives (i.e., if they talk about having a good teenage life without being enclosed by diabetes and are still well regulated).

- develop autonomously based motivation for blood glucose measurement, registration and regulation, because they think it is important and not because it is either imposed by parents/HCPs or driven by an "I should do" feeling.

- express their own goals for blood glucose and HbA1c regulation, and there is consistency between their objectives, values and behaviours.

- are conscious about what they want to talk about at the outpatient clinics.

- have insight into new ways to handle situations and relate constructively to the disease and their own reactions (e.g., instead of ignoring or deliberately choosing not to take insulin preventively, they now explain to their friends why they either opt out of eating certain foods or measure blood sugar and take insulin in advance.

- are able to communicate openly and honestly with parents and HCPs because there is an atmosphere where it is permissible and possible to be honest without experiencing condemnation.

- prevent or resolve conflicts or problems with diabetes in daily life outside the home and at home with support from parents and HCPs.

- are conscious about parents' and HCPs' resources and **s**eek advice from their parents and HCP when needed and take advantage of these resources in learning self-management of diabetes.

### Confidentiality

The study was approved by the Danish Data Association ref nr. 2008-41-2322. All information collected during the course of the study will be kept strictly confidential in accordance with Danish Data Association rules. The study will comply with all aspects of the Danish Data Association. Operationally, this will include consent from adolescents and parents to record the adolescents' personal details including name and date of birth and consent from adolescents and parents for the data collected for the study to be used to develop new research.

### Organization and Supervisors

A supervisory group comprising the co-authors of the present paper was established and is responsible for the project. The group will meet with the project leader (GRH) four times each year until the study is finished. The meetings will provide an opportunity to discuss the research design, methods for data collection, schedules, data analyses, outcomes and statistical challenges.

The day-to-day management of the study will be led by the project leader. Every week the project leader will meet with the involved HCPs who are running the intervention. These meetings will provide the opportunity to discuss current challenges regarding using the GSD-Y in routine outpatient clinical care.

## List of Abbreviations Used

HCP: health care providers; GSD-Y: guided self-determination - young; HbA1c: glycosylated haemoglobin

## Declaration of competing interests

The authors declare that they have no competing interests.

## Authors' contributions

All authors have read and approved the final manuscript. VZ is involved in the conception of the study and its implementation. VZ and BT have contributed to the design of the study and VZ, BT and BAE to development of the protocol. GRH drafted the manuscript with all authors providing critical review and final approval.

## Pre-publication history

The pre-publication history for this paper can be accessed here:

http://www.biomedcentral.com/1471-2431/11/55/prepub

## Supplementary Material

Additional file 1 **Appendix 1**. Content of GSD-Y training of paediatric diabetes HCPs. Click here for file

## References

[B1] Court JM Cameron FJ Berg-Kelly K Swift PG Diabetes in adolescence Pediatr Diabetes 2009 10 Suppl 12 185 194 19754629 10.1111/j.1399-5448.2009.00586.x

[B2] Bryden KS Dunger DB Mayou RA Peveler RC Neil HA Poor prognosis of young adults with type 1 diabetes: a longitudinal study Diabetes Care 2003 26 4 1052 1057 10.2337/diacare.26.4.1052 12663572

[B3] Laing SP Swerdlow AJ Slater SD Burden AC Morris A Waugh NR Mortality from heart disease in a cohort of 23,000 patients with insulin-treated diabetes Diabetologia 2003 46 6 760 765 10.1007/s00125-003-1116-6 12774166

[B4] Narayan KM Boyle JP Thompson TJ Sorensen SW Williamson DF Lifetime risk for diabetes mellitus in the United States JAMA 2003 290 14 1884 1890 10.1001/jama.290.14.1884 14532317

[B5] White NH Sun W Cleary PA Tamborlane WV Danis RP Hainsworth DP Effect of prior intensive therapy in type 1 diabetes on 10-year progression of retinopathy in the DCCT/EDIC: comparison of adults and adolescents Diabetes 2010 59 5 1244 1253 10.2337/db09-1216 20150283 PMC2857905

[B6] White NH Cleary PA Dahms W Goldstein D Malone J Tamborlane WV Beneficial effects of intensive therapy of diabetes during adolescence: outcomes after the conclusion of the Diabetes Control and Complications Trial (DCCT) J Pediatr 2001 139 6 804 812 11743505 10.1067/mpd.2001.118887

[B7] DCCT Effect of intensive diabetes management on the development and progression of long-term complications in adolescents with insulin dependent diabetes mellitus. Diabetes Control and Complications Trial J Pediatr 1994 125 2 177 188 8040759 10.1016/s0022-3476(94)70190-3

[B8] DCCT The effect of intensive treatment of diabetes on the development and progression of long-term complications in insulin dependent diabetes mellitus: Diabetes Control and Complications Trial. The Research Group N Engl J Med 1993 329 14 977 986 8366922 10.1056/NEJM199309303291401

[B9] Rewers M Pihoker C Donaghue K Hanas R Swift P Klingensmith GJ Assessment and monitoring of glycemic control in children and adolescents with diabetes Pediatr Diabetes 2007 8 6 408 418 10.1111/j.1399-5448.2007.00352.x 18036070

[B10] Helgeson VS Siminerio L Escobar O Becker D Predictors of metabolic control among adolescents with diabetes: a 4-year longitudinal study J Pediatr Psychol 2009 34 3 254 270 18667479 10.1093/jpepsy/jsn079 PMC2657034

[B11] Holmes CS Chen R Streisand R Marschall DE Souter S Swift EE Predictors of youth diabetes care behaviors and metabolic control: a structural equation modeling approach J Pediatr Psychol 2006 31 8 770 784 16221954 10.1093/jpepsy/jsj083

[B12] Dansk Register for Børne- og Ungdoms Diabetes.(DIA-REG B&U) Landsdækkende klinisk database for børn og unge med diabetes under 18 år Årsrapport 2009 1 60 2008/2009

[B13] Silverstein J Klingensmith G Copeland K Plotnick L Kaufman F Laffel L Care of children and adolescents with type 1 diabetes: a statement of the American Diabetes Association Diabetes Care 2005 28 1 186 212 10.2337/diacare.28.1.186 15616254

[B14] Lawson ML Sochett EB Chait PG Balfe JW Daneman D Effect of puberty on markers of glomerular hypertrophy and hypertension in IDDM Diabetes 1996 45 1 51 55 10.2337/diabetes.45.1.51 8522059

[B15] Helgeson VS Novak SA Illness centrality and well-being among male and female early adolescents with diabetes J Pediatr Psychol 2007 32 3 260 272 16837739 10.1093/jpepsy/jsl018

[B16] Suris JC Michaud PA Viner R The adolescent with a chronic condition. Part I: developmental issues Arch Dis Child 2004 89 10 938 942 10.1136/adc.2003.045369 15383438 PMC1719685

[B17] Kyngas H Barlow J Diabetes: an adolescent's perspective J Adv Nurs 1995 22 5 941 947 8568069 10.1111/j.1365-2648.1995.tb02646.x

[B18] Weinger K O'Donnell KA Ritholz MD Adolescent views of diabetes-related parent conflict and support: a focus group analysis J Adolesc Health 2001 29 5 330 336 10.1016/S1054-139X(01)00270-1 11691594 PMC1592605

[B19] Karlsson A Arman M Wikblad K Teenagers with type 1 diabetes -- a phenomenological study of the transition towards autonomy in self-management Int J Nurs Stud 2008 45 4 562 570 10.1016/j.ijnurstu.2006.08.022 17046768

[B20] Lewin AB Heidgerken AD Geffken GR Williams LB Storch EA Gelfand KM The relation between family factors and metabolic control: the role of diabetes adherence J Pediatr Psychol 2006 31 2 174 183 16467317 10.1093/jpepsy/jsj004

[B21] Graue M Wentzel-Larsen T Hanestad BR Sovik O Evaluation of a programme of group visits and computer-assisted consultations in the treatment of adolescents with Type 1 diabetes Diabet Med 2005 22 11 1522 1529 10.1111/j.1464-5491.2005.01689.x 16241917

[B22] Leonard BJ Garwick A Adwan JZ Adolescents' perceptions of parental roles and involvement in diabetes management J Pediatr Nurs 2005 20 6 405 414 10.1016/j.pedn.2005.03.010 16298281

[B23] Weissberg-Benchell J Nansel T Holmbeck G Chen R Anderson B Wysocki T Generic and diabetes-specific parent-child behaviors and quality of life among youth with type 1 diabetes J Pediatr Psychol 2009 34 9 977 988 10.1093/jpepsy/jsp003 19270028 PMC2782249

[B24] Anderson BJ Svoren B Laffel L Initiatives to promote effective self-care skills in children and adolescents with diabetes mellitus Dis Manage Health Outcomes 2007 15 2 101 108 10.2165/00115677-200715020-00005

[B25] Wiebe DJ Berg CA Korbel C Palmer DL Beveridge RM Upchurch R Children's appraisals of maternal involvement in coping with diabetes: enhancing our understanding of adherence, metabolic control, and quality of life across adolescence J Pediatr Psychol 2005 30 2 167 178 10.1093/jpepsy/jsi004 15681311

[B26] Dunger DB Williams RM The challenging years: surviving adolescence Int J Clin Pract 2003 11 Suppl 23 29

[B27] Wysocki T Greco P Social support and diabetes management in childhood and adolescence: influence of parents and friends Curr Diab Rep 2006 6 2 117 122 10.1007/s11892-006-0022-y 16542622

[B28] Ivey JB Wright A Dashiff CJ Finding the balance: adolescents with type 1 diabetes and their parents J Pediatr Health Care 2009 23 1 10 18 10.1016/j.pedhc.2007.12.008 19103402

[B29] Dashiff C Hardeman T McLain R Parent-adolescent communication and diabetes: an integrative review J Adv Nurs 2008 62 2 140 162 10.1111/j.1365-2648.2007.04549.x 18394028

[B30] Weissberg-Benchell J Antisdel JE Balancing developmental needs and intensive management in adolescents Diabetes Spectr 2000 13 2 88 94

[B31] Hoey H Hvidoere Study Group on Childhood Diabetes Psychosocial factors are associated with metabolic control in adolescents: research from the Hvidoere Study Group on Childhood Diabetes Pediatr Diabetes 2009 10 Suppl 13 9 14 19930221 10.1111/j.1399-5448.2009.00609.x

[B32] Wysocki T Nansel TR Holmbeck GN Chen R Laffel L Anderson BJ Collaborative involvement of primary and secondary caregivers: associations with youths' diabetes outcomes J Pediatr Psychol 2009 34 8 869 881 10.1093/jpepsy/jsn136 19112077 PMC2729681

[B33] Viklund G Wikblad K Teenagers' perceptions of factors affecting decision-making competence in the management of type 1 diabetes J Clin Nurs 2009 18 23 3262 3270 10.1111/j.1365-2702.2009.02963.x 19930085

[B34] Olinder AL Self-management of diabetes in adolescents using insulin pumps PhD thesis 2010 Uppsala Universitet, Uppsala, Acta Universitatis Upsaliensis

[B35] Corlett J Twycross A Negotiation of parental roles within family-centred care: a review of the research J Clin Nurs 2006 15 10 1308 1316 10.1111/j.1365-2702.2006.01407.x 16968435

[B36] Butler DA Zuehlke JB Tovar A Volkening LK Anderson BJ Laffel LM The impact of modifiable family factors on glycemic control among youth with type 1 diabetes Pediatr Diabetes 2008 9 4 Pt 2 373 381 18774997 10.1111/j.1399-5448.2008.00370.x

[B37] Jones K Hammersley S Shepherd M Meeting the needs of young people with diabetes: an ongoing challenge J Diabetes Nurs 2003 7 9 345 350

[B38] Williams C Gender, adolescence and the management of diabetes J Adv Nurs 1999 30 5 1160 1166 10.1046/j.1365-2648.1999.01168.x 10564415

[B39] Luyckx K Seiffge-Krenke I Continuity and change in glycemic control trajectories from adolescence to emerging adulthood: relationships with family climate and self-concept in type 1 diabetes Diabetes Care 2009 32 5 797 801 10.2337/dc08-1990 19228859 PMC2671120

[B40] Anderson BJ Holmbeck G Iannotti RJ McKay SV Lochrie A Volkening LK Dyadic measures of the parent-child relationship during the transition to adolescence and glycemic control in children with type 1 diabetes Fam Syst Health 2009 27 2 141 152 19630455 10.1037/a0015759 PMC2843423

[B41] Michaud PA Suris JC Viner R The adolescent with a chronic condition. Part II: healthcare provision Arch Dis Child 2004 89 10 943 949 10.1136/adc.2003.045377 15383439 PMC1719690

[B42] Hanna KM Guthrie D Adolescents' behavioral autonomy related to diabetes management and adolescent activities/rules Diabetes Educ 2003 29 2 283 291 10.1177/014572170302900219 12728755

[B43] Murphy HR Wadham C Rayman G Skinner CT Integrating pediatric diabetes education into routine clinical care: the Families, Adolescents and Children's Teamwork Study (FACTS) Diabetes Care 2006 29 5 1177 1177 10.2337/dc06-0259 16644662

[B44] Delamater AM Psychological care of children and adolescents with diabetes Pediatr Diabetes 2009 10 Suppl 12 175 184 19754628 10.1111/j.1399-5448.2009.00580.x

[B45] Grey M Boland EA Davidson M Li J Tamborlane WV Coping skills training for youth with diabetes mellitus has long-lasting effects on metabolic control and quality of life J Pediatr 2000 137 1 107 113 10.1067/mpd.2000.106568 10891831

[B46] Laffel LM Vangsness L Connell A Goebel-Fabbri A Butler D Anderson BJ Impact of ambulatory, family-focused teamwork intervention on glycemic control in youth with type 1 diabetes J Pediatr 2003 142 4 409 416 10.1067/mpd.2003.138 12712059

[B47] Zoffmann V Lauritzen T Guided self-determination improves life skills with Type 1 diabetes and A1C in randomized controlled trial Patient Educ Couns 2006 64 1-3 78 86 10.1016/j.pec.2005.11.017 16720089

[B48] Creswell JW Clark VLP Designing and Conducting Mixed Methods Research 2007 Thousand Oaks, CA: Sage Publications

[B49] Zoffmann V Kirkevold M Life versus disease in difficult diabetes care: conflicting perspectives disempower patients and professionals in problem solving Qual Health Res 2005 15 6 750 765 10.1177/1049732304273888 15961873

[B50] Zoffmann V Kirkevold M Relationships and their potential for change developed in difficult type 1 diabetes Qual Health Res 2007 17 5 625 638 10.1177/1049732307301230 17478645

[B51] Zoffmann V Harder I Kirkevold M A person-centered communication and reflection model: sharing decision-making in chronic care Qual Health Res 2008 18 5 670 685 10.1177/1049732307311008 18223158

[B52] Mullen D A Conceptual Framework for the Life Skills Program 1985 Toronto, The Guidance Centre, University of Toronto

[B53] Brooks DK A life-skills taxonomy: defining elements of effective functioning through the use of delphi technique 1984 PhD thesis. University of Georgia

[B54] Anderson B Funnell MM The Art of Empowerment: Stories and Strategies for Diabetes Educators 2000 American Diabetes Association

[B55] Deci EL Ryan RM Intrinsic Motivation and Self-Determination in Human Behavior 1985 New York: Plenum Press

[B56] Ryan RM Deci EL Self-determination theory and the facilitation of intrinsic motivation, social development, and well-being Am Psychol 2000 55 1 68 78 11392867 10.1037//0003-066x.55.1.68

[B57] Nutbeam D Health promotion glossary Health Promot Int 1998 13 4 349 364 10.1093/heapro/13.4.349 16963461

[B58] Niemiec CP Lynch MF Vansteenkiste M Bernstein J Deci EL Ryan RM The antecedents and consequences of autonomous self-regulation for college: a self-determination theory perspective on socialization J Adolesc 2006 29 5 761 775 10.1016/j.adolescence.2005.11.009 16412502

[B59] Bos AH [The Model of Dynamic Judgement Building] Urteilsbuilding in Gruppen: Polarität und Rhytmus als Schüssel zur Entwicklung sozialer OrganismenInstitut für Sozialforschung, Praxisberatung und Organizationsentwicklung 2001 PhD thesis. Deutschland

[B60] Steinberg JM Andresen AF Aktivt verdivalg: Meninger og handlinger: En pedagogisk metodikk. 1. udgave, 2. oplag ed 1981 Oslo: Dreyer

[B61] Prochaska JO Norcross JC DiClemente CC Changing for Good 2002 New York: Quill (Reprinted)

[B62] Clabby J O'Connor R Teaching learners to use mirroring: rapport lessons from neurolinguistic programming Fam Med 2004 36 8 541 543 15343412

[B63] Gordon T Kragh B Forældreuddannelse: problemer, konflikter, løsninger. 3. udgave ed 1999 Valby: Borgen

[B64] Faber A Mazlish E Om at tale så mine børn lytter - og om at lytte så de taler 1993 Forum, København, Danmark [Kbh.]: Forum

[B65] Williams GC Freedman ZR Deci EL Supporting autonomy to motivate patients with diabetes for glucose control Diabetes Care 1998 21 10 1644 1651 10.2337/diacare.21.10.1644 9773724

[B66] Williams GC McGregor HA Zeldman A Freedman ZR Deci EL Testing a self-determination theory process model for promoting glycemic control through diabetes self-management Health Psychol 2004 23 1 58 66 14756604 10.1037/0278-6133.23.1.58

[B67] Polonsky WH Anderson BJ Lohrer PA Welch G Jacobson AM Aponte JE Assessment of diabetes-related distress Diabetes Care 1995 18 6 754 760 10.2337/diacare.18.6.754 7555499

[B68] Robbins RJ An assessment of perceptions of parental autonomy support and control: child and parent correlates. Unpublished Doctoral Dissertation, Department of Psychology, University of Rochester 1994

[B69] de Wit M Pouwer F Gemke RJ Delemarre-van de Waal HA Snoek FJ Validation of the WHO-5 Well-Being Index in adolescents with type 1 diabetes Diabetes Care 2007 30 8 2003 2006 10.2337/dc07-0447 17475940

[B70] Kvamme OJ Mainz J Helin A Ribacke M Olesen F Hjortdahl P Oversettelse av spørreskjema: et oversett metodeproblem Nordisk Medicin 1998 113 10 363 366 9894417

[B71] Wagner JA Response shift and glycemic control in children with diabetes Health Qual Life Outcomes 2005 3 38 10.1186/1477-7525-3-38 15955236 PMC1180844

[B72] Malterud K Kvalitativa metoder i medicinsk forskning: en introduktion 2009 Lund: Studentlitteratur

[B73] Gilchrist LD Schinke SP Maxwell JS Life skills counseling for preventing problems in adolescence J Soc Serv Res 1987 10 2-4 73 84

[B74] Sidani S Doran DM Mitchell PH A theory-driven approach to evaluating quality of nursing care J Nurs Scholarsh 2004 36 1 60 65 10.1111/j.1547-5069.2004.04014.x 15098420

[B75] Glaser BG Theoretical Sensitivity: Advances in the Methodology of Grounded Theory 1978 Mill Valley, CA: Sociology Press

