# Supplementary material for: Improving glycaemic control and life skills in adolescents with type 1 diabetes: A randomised, controlled intervention study using the Guided Self-Determination-Young method in triads of adolescents, parents and health care providers integrated into routine paediatric outpatient clinics
Source: BMC Pediatr. 2011 Jun 14;11:55. doi: 10.1186/1471-2431-11-55 (PMC3164223; doi:10.1186/1471-2431-11-55)
Supplement: Additional file 1 — Appendix 1. Content of GSD-Y training of paediatric diabetes HCPs. [file 1471-2431-11-55-S1.PDF]

Content of GSD-Y training of paediatric diabetes HCPs

- 10 1-hour sessions conveying the theoretical idea of GSD adjusted to GSD-Y
- 20 1-hour sessions comprising communication theory and practising the use of reflection sheets in fictive cases under supervision of GRH and VZ
- 20 1-hour sessions training in communication skills in dialogue between three parties in fictive cases
- Practising the use of GSD-Y reflection sheets in interactions with 2 adolescents with type 1 diabetes and their parents in outpatient visits under supervision from GRH and VZ
- Recording outpatient appointments from each HCP to ensure that the GSD-Y is used correctly, checked by GRH and VZ
- Written and oral tests in the formal theoretical foundation of GSD
